# Supplementary material for: Common Variable Immunodeficiency: A Standardized Patient Case for Second-Year Medical Students
Source: MedEdPORTAL. 2019 Oct 18;15:10837. doi: 10.15766/mep_2374-8265.10837 (PMC6974347; doi:10.15766/mep_2374-8265.10837)
Supplement: Supplementary file 1 — A. SP Case.docx B. SP Training Notes.docx C. PE Cards.docx D. Moulage.docx E. Door Chart and Instructions.docx F. Postencounter and Rubric.docx G. SP Checklist.docx [file mep-15-10837-s001.zip › _Educational_Summary_Report_10837.pdf]

MedEdPORTAL® The AAMC Journal of Teaching and Learning Resources

Original Publication Open Access

Common Variable Immunodeficiency: A Standardized Patient Case for Second-Year Medical Students

Maria-Louise Barilla-LaBarca, MD*, Monica Rodriguez, Kelly Connors, Theresa Wanamaker, MS, Marie Cavuoto Petrizzo, MD

*Corresponding author: mbarilla@northwell.edu

Abstract

Introduction: Common variable immunodeficiency (CVID) is the most common symptomatic antibody deficiency, with a prevalence of 0.6-6.9 depending on the population studied. In contrast to other primary immunodeficiency diseases (PIDDs), symptoms may not appear until the third decade of life. Lack of recognition of CVID is a persistent problem. Myriad confounding clinical phenotypes and frequent infections, including autoimmunity, malignancy, chronic lung disease, granulomatous disease, and gastrointestinal disease, complicate the diagnosis. Often it is years before a diagnosis is made, leading to irreversible morbidities and mortality. **Methods:** Second-year medical students are introduced to CVID during their session on PIDDs that occurs during the immunology/rheumatology course. To assess students' recognition of CVID, a 15-minute OSCE encounter was created that included a simulation of lung sounds (rhonchi), physical exam cards (clubbing, otitis media with effusion), and moulage of skin (petechiae). A standardized patient (SP) portrayed a patient requesting antibiotics for a sinus infection. Students were tasked to both interview the patient and perform a hypothesis-driven physical exam. A postencounter exercise queried the students on their differential diagnosis and their rationale. **Results:** Item analysis of the case showed high levels of difficulty and strong discrimination between high- and low-performing students in both communication skills and clinical reasoning in CVID. **Discussion:** This SP encounter can be used in both formative and summative assessments to measure the recognition of CVID.

Keywords
Primary Immunodeficiency, Common Variable Immunodeficiency, OSCE, Immunology, Allergy and Immunology, Rheumatology, Clinical Skills Assessment

Educational Objectives

By the end of this activity, learners will be able to:

1. Obtain a focused history for a patient presenting with recurrent sinopulmonary infections.
2. Recognize a primary immunodeficiency as an underlying cause of frequent infections in adults.
3. Perform a hypothesis-driven examination in a patient presenting with frequent sinopulmonary infections.
4. Generate an appropriate differential in a patient with recurrent sinopulmonary infections and petechial rash.

Citation:
Barilla-LaBarca M-L, Rodriguez M, Connors K, Wanamaker T, Cavuoto Petrizzo M. Common variable immunodeficiency: a standardized patient case for second-year medical students. *MedEdPORTAL*. 2019;15:10837. https://doi.org/10.15766/mep_2374-8265.10837

Introduction

Common variable immunodeficiency (CVID) is the most common symptomatic antibody deficiency, with a prevalence range of 0.6-6.9 depending on the population studied.[1] In contrast to other primary immunodeficiency diseases (PIDDs), symptoms may not appear until young adulthood, typically the third decade of life.[2] Lack of recognition remains a persistent problem. Myriad confounding clinical phenotypes and frequent infections, including autoimmunity, malignancy, chronic lung disease, granulomatous disease, and gastrointestinal disease, complicate the diagnosis. A recent publication noted a mean diagnostic delay of almost 9 years for patients with CVID, which can lead to irreversible morbidities and mortality.[3]

Second-year medical students are introduced to CVID during a 2-hour large-group session on PIDDs that occurs during the immunology/rheumatology course. To assess students' recognition of CVID and their skill at taking a medical history in a patient with signs suggestive of CVID, a 15-minute standardized patient (SP) encounter was created that evaluated

Copyright © 2019 Barilla-LaBarca et al. This is an open-access publication distributed under the terms of the Creative Commons Attribution license.

communication, history, physical exam, professionalism, and clinical reasoning. An SP-driven assessment was chosen rather than other types of knowledge tests for several reasons, including the potential to obtain a rich medical history, the chance to assess if students utilized pertinent hypothesis-driven questions to elicit the correct information (as opposed to recognizing the patterns of PIDD when clearly stated), and ample opportunity to allow students to express their clinical reasoning. This encounter was incorporated in the students' high-stakes summative OSCE at the end of their course.

To our knowledge, there are neither formative nor summative SP-based assessments of PIDD in *MedEdPORTAL* or in the literature. This SP case therefore contributes to the existing literature a tool that can be useful in curricular pushout, assessment, or skill building. Furthermore, the case is adaptable to many learner levels.

Methods

The target audience was preclinical medical students enrolled in an immunology course; however, this case could be extended without adaptation to other learner levels. Learners engaged in one large-group didactic session directly addressing PIDDs, including CVID, in the context of a 7-week course covering basic immunology and rheumatology. Learning objectives included recognition of PIDDs of the adaptive and innate immune system, identification and interpretation of pertinent diagnostic testing, and understanding the implications of missing these diagnoses on morbidity and mortality.

Following the course, as with all other courses at our institution, students participated in a six-station summative OSCE that covered topics in rheumatology and immunology. The OSCE took place in a clinical skills/simulation center. Each encounter was 15 minutes in length, followed by a 10-minute postencounter. This case (Appendix A) represented one of those six cases. The other cases addressed muscle weakness (one station was history and the other was physical exam), inflammatory small joint pain, noninflammatory shoulder pain (physical exam), and adolescent medicine interviewing skills. To assess the learning objective of recognizing PIDD, a case on CVID was developed. CVID was chosen as the subject for the PIDD case not only because of its high prevalence in adults but also because its phenotypic presentation allowed for the obtainability of a robust medical history. Furthermore, there was ample opportunity for students to demonstrate their aptitude in clinical reasoning as they distinguished between rheumatologic, immunologic, and infectious diseases.

Recruitment of SPs for this case was determined by the clinical skills center's trainers and targeted men or women in their third or fourth decade of life. Between four and six SPs were recruited for each case depending on the rotation schedule and to allow for breaking between students. Each SP would be in role between four and seven times in a day's event.

Two trainings of 3 hours each were given, the typical format of training for all summative encounters at our institution. During the first training, two authors (Maria-Louise Barilla-LaBarca and Marie Cavuoto Petrizzo) were present in addition to the assigned SP trainer to assist with review of the SP training notes (Appendix B), checklist items (Appendix G), and moulage testing and approval (Appendix D).

Portrayal feedback and accuracy of checklist scoring were accomplished at several checkpoints. Each training session was led by a trainer who was an experienced SP and who was prepared to provide portrayal feedback to SPs during role-play and checklist item feedback during group debrief. On the day of the OSCE event, trainers observed cases via video live recording and corrected in real time any issues that arose. Furthermore, select stations were viewed by quality assurance SPs who also filled out checklists that could be compared for accuracy following the event. Finally, random videos were reviewed by faculty postevent for purposes of scoring students, but any issues with portrayal or checklist accuracy were noted, discussed with the clinical skills team, and annotated for the following year.

Moulage was applied by SP trainers (Monica Rodriguez and Kelly Connors) using a lightly moistened porous sea sponge and applicator as described in Appendix D. Color palette and hue were adjusted for skin tone (Figures 1-3). Appearance was shown, modified, and given final approval by three clinicians, including a rheumatologist (Maria-Louise Barilla-LaBarca), immunologist (Marie Cavuoto Petrizzo), and dermatologist (faculty at our school).

The simulated setting was an urgent care facility visit. A door chart (Appendix E) provided students with the patient's name, date of birth, and vital signs. It also instructed the students to obtain relevant history and perform an examination. When ready, the student entered the examination room and conducted a history and physical. The SP, when asked, stated a chief complaint of needing "antibiotics for yet another sinus infection." The SP reported frequent sinopulmonary infections over several years as an adult and carried a recent diagnosis of bronchiectasis. Additionally, there were some vague gastrointestinal complaints and the recent onset of a petechial

Copyright © 2019 Barilla-LaBarca et al. This is an open-access publication distributed under the terms of the Creative Commons Attribution license.

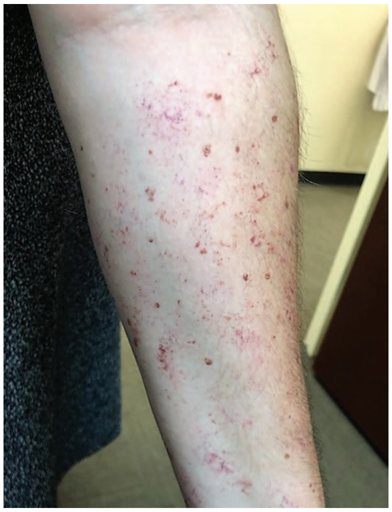

Figure 1. Petechiae moulage on light skin tone.

rash. Students were given a simulated stethoscope (SimScope; Cardionics, Webster, Texas) to evaluate the lungs and heart, which were programmed for rhonchi and normal heart sounds. Physical exam cards (clubbing, otitis media with effusion) were given to the students only if they examined the specific body part (Appendix C). Finally, a petechial rash was portrayed using moulage (Appendix D). The students had the entire 15 minutes to perform a history and physical and were given a 5-minute warning to help them pace themselves.

Learner Assessment

SPs completed checklists (Appendix G). These 27-item checklists contained items related to communication skills, physical

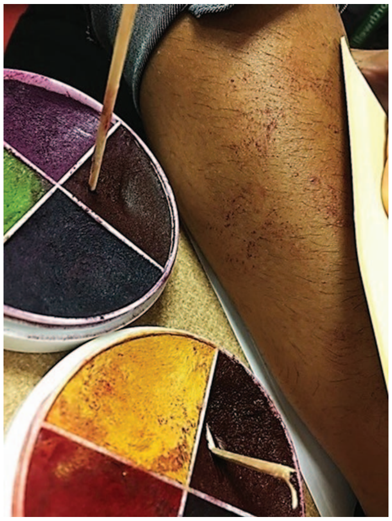

Figure 2. Petechiae moulage on medium skin tone.

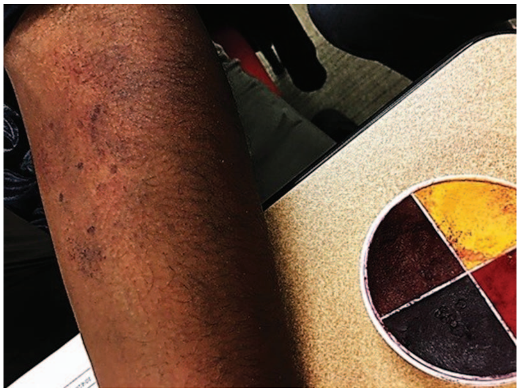

Figure 3. Petechiae moulage on dark skin tone.

diagnosis, professionalism, and clinical reasoning. Many of the questions were standard ones that appeared on all OSCEs at our institution and reflected our longitudinal communication, professionalism, and physical exam curricula.[4] SPs received annual and quarterly training with feedback specific to these questions. The clinical reasoning questions addressed specific historical questions related to PIDDs, as well as what the course director and content expert considered a relevant hypothesis-driven physical exam.[5,6] Those questions that were specific to this case were developed by the course director, who was also the medical director of clinical skills (Maria-Louise Barilla-LaBarca) and who had, in that capacity, experience in generating SP checklists. To enhance accuracy, all checklist items were anchored.

When students completed the encounter, they left the room and answered a postencounter question on a computer (Appendix F) related to the differential diagnosis and rationale. Up to 10 minutes was allowed for this postencounter question.

Following the examination, the postencounter question was graded on a preset rubric by a single faculty member (Appendix F). The ideal answer was written concurrently with the case to ensure validity. The preset rubric was then developed by the facilitator of the session (Marie Cavuoto Petrizzo), who was the sole rater, and the course director (Maria-Louise Barilla-LaBarca) based on the ideal answer and other similar clinical reasoning questions that occurred in our clinical skills center.

Effectiveness of the test questions was determined through a standard item analysis by the assessment office and included a measurement of the difficulty and the discrimination of each question. Item difficulty was measured as the percentage of students who got the item correct. Item discrimination was used to assess whether the question could distinguish between

Copyright © 2019 Barilla-LaBarca et al. This is an open-access publication distributed under the terms of the Creative Commons Attribution license.

students who knew the material and those who did not and was calculated by finding the difference between the score in the upper quartile of students and the score in the lower quartile of students. The higher the percentage, the better the question was as a discriminator, with values above 25% looked upon favorably. The analysis was performed on both individual checklist items and the postencounter question to determine if this assessment was appropriately challenging and if it could discriminate between high- and low-performing students.

There was no passing score for this case, as the checklist from all six cases was subdivided into different competency-based domains and pooled. Students therefore could not receive a failing score based on their performance in this one case. Videos from students performing in the lowest standard deviation of the class in each domain were reviewed by the clinical skills director. Findings were presented for discussion with the assessment team of the school of medicine to arrive at a final domain score. These final domain scores were then evaluated by the assessment team by preset standards that differed per course and per learner level to determine if the student met the minimum standard to pass the OSCE. Checklist scores, postencounter grades, and videotapes were available to every student following the examination, and any areas of weakness could be addressed by faculty on request.

Results

All second-year students enrolled in the immunology/rheumatology course Interacting With the Environment took the final summative OSCE assessment ($N = 102$), including this encounter. Item analysis (Table) showed high levels of discrimination in both communication skills and clinical reasoning. As expected, checklist items related to performance of the physical exam and history taking were not difficult and not discriminating. Three clinical reasoning questions (childhood infections, examination of the spleen, and inspection of nailbeds) and two communication questions (empathy and impact/concern/explanatory model) posed higher levels of difficulty, with less than 50% of the class attaining a correct score. One clinical reasoning question (examination of lungs) displayed 0% discrimination. Similarly, the postencounter question demonstrated a high level of difficulty and was highly discriminating between students (Table). These results were concordant with the item analysis for the other OSCE cases and aligned with our expectation of performance for this learner level.

Discrimination statistics of the postencounter question using the performance on the final immunology essay as a surrogate marker of understanding of immunologic principles in a non–SP-driven written assessment showed similar discrimination—that is, students who performed well on the postencounter question performed well overall on the written examination (29% discrimination). Our final essay could be subdivided into three types of questions: first order, application, and problem solving. Discrimination between students based on the problem-solving score was slightly higher than the first-order or application score, although they all were below 11%.

Discussion

Although SP assessments have proven to be helpful in both formative and summative assessments, their utilization in encounters pertaining to PIDDs has not yet been reported. This case is easily portrayed and executed. Moulage was easily applied, was durable throughout the day of testing, and contributed to the clinical reasoning in the case. In addition to the assessment of students, this encounter can be used by educators to identify curricular weaknesses and educational gaps relative to their teaching material.

The two items in clinical reasoning that had low discrimination were performance of a lung examination and the elicitation of history of frequent infection. This can be explained by SimScope placement in the room, which biased students to perform a lung examination. Therefore, the question will be removed from future assessments. Furthermore, on review of script portrayal, the history of multiple infections came out very naturally and obviously as part of the backstory without students' specific inquiry. Thus, eliciting this historical element may be measuring not clinical reasoning skills but rather the ability to ask an open-ended question early in the history of present illness. For this reason, this item could be removed from the checklist or the SPs could be coached to not give up the history of frequent infections as readily. Importantly, items with high levels of difficulty alerted our faculty to potential weakness in the curricular pushout of this material and are being revisited as plans for the next academic year take place. For example, the question on inquiry into childhood infections was difficult for the entire class, with relatively low discrimination, suggesting that weakness in this area was a curricular issue. Next year, more emphasis on this through case-based vignettes with rich histories may be added.

This case was appropriately and expectedly challenging for our medical students. Although students displayed strong communication skills during the encounter, they performed less well on empathy. This had been anecdotally observed in our students in the past, where empathy skills were lower in

Copyright © 2019 Barilla-LaBarca et al. This is an open-access publication distributed under the terms of the Creative Commons Attribution license.

Table. Checklist and Postencounter Item Analysis ($N = 102$)

Question	Average	Full Credit (%)	Zero Credit (%)	Difficulty (%)	Discrimination (%)
Did the student refrain from exhibiting any distracting nonverbal behaviors?	1.94	97.06	2.94	97.06	8
Did the student use the skills of empathy in relation to an expressed or potential feeling/emotion?	1.16	34.31	18.63	57.84	44
Did the student elicit the impact on your life, your concerns, or your explanatory model?	1.20	45.10	25.49	59.80	50
Did the student ask your chief complaint?	2.00	100	0	100	0
Did the student use medical jargon without explaining it?	1.84	84.31	0	92.16	8
Did the student review and summarize the gathered information from the history of present illness?	1.53	76.47	23.53	76.47	36
Did the student sanitize his or her hands with soap and water, or Purell, before any physical contact?	1.81	82.35	0.98	90.69	22
Did the student attend to your comfort and modesty during the physical exam?	1.84	84.31	0	92.16	8
Did the student update you as to what to expect during the physical exam?	2.00	100	0	100	0
Was the progression of the physical exam fluid?	1.97	97.06	0	98.53	0
Did the student perform all physical exam procedures on skin?	1.80	82.35	1.96	90.20	4
Did the student elicit that you have a long history of infections?	1.78	89.22	10.78	89.22	4
Did the student ask about infections during your childhood?	0.71	35.29	64.71	35.29	16
Did the student ask if you are short of breath?	1.12	55.88	44.12	55.88	16
Did the student examine your skin besides your hands?	1.39	69.61	30.39	69.61	32
Did the student examine your nose?	1.20	59.80	40.20	59.80	40
Did the student look in your ears?	1.63	81.37	18.63	81.37	36
Did the student look in your mouth?	1.39	69.61	30.39	69.61	28
Did the student examine any of your lymph nodes?	1.16	57.48	42.16	57.84	24
Did the student examine your lungs?	2.00	100	0	100	0
Did the student examine your spleen?	0.35	17.65	82.35	17.65	36
Did the student examine your nails?	0.53	26.47	73.53	26.47	24
Postencounter	5.46	1.96	0	49.64	29

encounters that had higher cognitive load, and deserves further study. The favorable item analysis, with appropriate levels of difficulty and a high level of discrimination in most questions, supports its continued use as a summative assessment.

The skin finding of petechiae was an important distinguishing feature of the case. Students would have to examine the skin to interpret it. The SPs were instructed to note how flat it felt, which would provide an important diagnostic clue to prioritizing the differential diagnosis appropriately.

One limitation to the execution of this case is the hybrid nature of the physical exam, which utilizes SimScope technology in depicting abnormal lung sounds and is not available at all institutions. Although this technology offers students the ability to listen to abnormal lung sounds and is our preferred method, neither the portrayal of the patient nor the development of the differential depends on it. Considering that this patient already carries a diagnosis of bronchiectasis, the identification of abnormal lung sounds is critical not to the clinical reasoning of the case but rather to the overall fidelity of the patient's story. A physical exam card could be used to describe the pulmonary findings or an audio file clip could be given to the student after the encounter (if interpretation is important to the educator). A second limitation to using the SimScope occurred after several of our SPs developed a skin irritation from the pads of the SimScope after the first day. Pad placement was thereafter placed on a picture of a patient's posterior and anterior chest.

Students were not queried about their reactions to the case or their impression of fidelity with hybrid simulations, as this was a summative examination. Students were given an opportunity to provide comments on the OSCE experience at the end of every course, although not on any case individually.

Although we chose to use an OSCE to assess knowledge on CVID, other assessments could have also measured students' understanding of the concepts presented in the didactic session. Complex SP cases such as this certainly add a level of difficulty over traditional medical knowledge testing, as they require the student to solicit relevant information as opposed to recognizing it. Therefore, the scores may underestimate the actual ability to recognize patterns of disease and diagnostic accuracy.

The examination occurred at the beginning of assessment week, and therefore, one potential barrier to success on this case was that students may have needed more time to study. To offset this potential barrier, a formative simulation experience on PIDD led by clinical immunologists preceded the event for most of the class.

Copyright © 2019 Barilla-LaBarca et al. This is an open-access publication distributed under the terms of the Creative Commons Attribution license.

In the future, we plan to continue to use this scenario in the high-stakes summative final OSCE for students in this course. We plan to continue to use data from this scenario to contribute to the overall clinical skills grade of the student. Additionally, we will use the data to assist our clinical immunology faculty in modifying the PIDD didactic session, targeting areas of student deficits on the OSCE that thus require further emphasis. Given that recognition of this disorder is difficult, even when the subject matter is fresh in learners' minds, we will continue to modify PIDD sessions using this case as a benchmark to guide us. Although we developed this scenario for second-year medical students learning basic immunology, the case is robust enough to offer an appropriate challenge for higher-level learners because it parallels clinical practice, where there is a known gap in recognition of CVID. Such a case measures the recognition of CVID, which is a critical first step in providing lifesaving care to these patients.

Appendices

A. SP Case.docx
B. SP Training Notes.docx
C. PE Cards.docx
D. Moulage.docx
E. Door Chart and Instructions.docx
F. Postencounter and Rubric.docx
G. SP Checklist.docx

All appendices are peer reviewed as integral parts of the Original Publication.

Maria-Louise Barilla-LaBarca, MD: Associate Professor, Department of Medicine/Division of Rheumatology, Donald and Barbara Zucker School of Medicine at Hofstra/Northwell; Co-Course Director, Interacting With the Environment, Donald and Barbara Zucker School of Medicine at Hofstra/Northwell

Monica Rodriguez: Standardized Patient Educator, Clinical Skills Center, Northwell Health

Kelly Connors: Standardized Patient Educator, Clinical Skills Center, Northwell Health

Theresa Wanamaker, MS: Manager, Clinical Skills Center, Northwell Health

Marie Cavuoto Petrizzo, MD: Assistant Professor, Department of Science Education, Donald and Barbara Zucker School of Medicine at Hofstra/Northwell; Assistant Professor, Department of Pediatrics, Donald and Barbara Zucker School of Medicine at Hofstra/Northwell; Assistant Course Director, Patient, Physician and Society, Donald and Barbara Zucker School of Medicine at Hofstra/Northwell

Acknowledgments

We would like to thank Wendy Herman, MLIS, AHIP (Head of Education and Access Services and Assistant Professor of Science Education), for her help in securing permissions for images. We also would like to thank the Clinical Skills Center standardized patients for their hard work and dedication.

Disclosures

None to report.

Funding/Support

None to report.

Ethical Approval

Reported as not applicable.

References

1. Selenius JS, Martelius T, Pikkarainen S, et al. Unexpectedly high prevalence of common variable immunodeficiency in Finland. *Front Immunol.* 2017;8:1190. https://doi.org/10.3389/fimmu.2017.01190
2. Bergbreiter A, Salzer U. Common variable immunodeficiency: a multifaceted and puzzling disorder. *Expert Rev Clin Immunol.* 2009;5(2):167-180. https://doi.org/10.1586/1744666X.5.2.167
3. Quinti I, Soresina A, Spadaro G, et al. Long-term follow-up and outcome of a large cohort of patients with common variable immunodeficiency. *J Clin Immunol.* 2007;27(3):308-316. https://doi.org/10.1007/s10875-007-9075-1
4. Cole SA, Bird J. *The Medical Interview: The Three Function Approach*. 3rd ed. Philadelphia, PA: Elsevier Saunders; 2014.
5. Garibaldi BT, Olson APJ. The hypothesis-driven physical examination. *Med Clin North Am*. 2018;102(3):433-442. https://doi.org/10.1016/j.mcna.2017.12.005
6. Yudkowsky R, Otaki J, Bordage G, Lowenstein T, Riddle J, Nishigori H. Hypothesis-Driven Physical Examination student handbook. *MedEdPORTAL*. 2011;7:8294. https://doi.org/10.15766/mep_2374-8265.8294

Received: January 19, 2019
Accepted: June 5, 2019
Published: October 11, 2019

Copyright © 2019 Barilla-LaBarca et al. This is an open-access publication distributed under the terms of the Creative Commons Attribution license.
