# Supplementary material for: Common Variable Immunodeficiency: A Standardized Patient Case for Second-Year Medical Students
Source: MedEdPORTAL. 2019 Oct 18;15:10837. doi: 10.15766/mep_2374-8265.10837 (PMC6974347; doi:10.15766/mep_2374-8265.10837)
Supplement: Supplementary file 1 — A. SP Case.docx B. SP Training Notes.docx C. PE Cards.docx D. Moulage.docx E. Door Chart and Instructions.docx F. Postencounter and Rubric.docx G. SP Checklist.docx [file mep-15-10837-s001.zip › A. SP Case.docx]

Appendix A:  *SP Case*

**Date:** Developed August 2018, Executed October 2018

**Primary Case Author:** Maria-Louise Barilla-LaBarca, MD

**Secondary Case Author:**  Marie Cavuoto Petrizzo, MD

**Standardized Patient Educator:** Monica Rodriguez, BFA

**Name of Case:**  Robin Samuels

**Name of educational activity:** Final OSCE assessment

**Patient Name:** Robin Samuels

**Chief Complaint:**  “I’m here to get antibiotics for my sinus infection”

**Most Likely Diagnosis and Differential with rationale from history and/or physical exam:**

1. Sinusitis due to underlying primary immunodeficiency (common variable immunodeficiency)
   1. historical support:
      1. recurrent sinopulmonary infections that began after childhood
      2. poor response to immunizations
      3. bronchiectasis
      4. diarrhea
      5. increased bruising (for associated autoimmune ITP)
   2. pe support:
      1. otitis media (in adult),
      2. clubbing (in support of bronchiectasis)
      3. purpura (possibly due to ITP as it blanches without palpable component making vasculitis much less likely).
2. Sinus and pulmonary disease from vasculitis (Granulomatosis with polyangiitis)
   1. historical support:
      1. systemic disease, affecting multiple organs
      2. characteristic pattern of involvement upper respiratory and lower pulmonary disease
      3. history of rash
   2. pe support:
      1. Otitis media (in adult),
      2. petechial/purpuric rash
3. Sinusitis from disorders that cause impaired airway clearance (cystic fibrosis and ciliary dyskinesia)
   1. historical support:
      1. recurrent sinopulmonary infections
      2. chronic purulent cough with bronchiectasis
   2. pe support:
      1. Otitis media (in adult)

Challenge question: none

Domains:

🗹 Professionalism

🗹 Communication and Interpersonal skills

🗹 Medical History

🗹 Physical exam

- Shared Decision Making
- Patient Education

🗹 Clinical Reasoning

- Documentation
- Handoff
- Presentation
- Other:

Type and level of Learner: Medical Student, Preclinical

Case Objectives

Primary objectives - clinical reasoning:

- Obtain a focused history for a patient presenting with recurrent sinopulmonary infections
- Recognize a primary immunodeficiency as an underlying cause of frequent infections in adults
- Perform a hypothesis driven examination in a patient presenting with frequent sinopulmonary infections.
- Generate an appropriate differential in a patient with recurrent sinopulmonary infections and petechial rash

Secondary objectives:

Professionalism:

- Assess a learner’s professionalism with patients.

Communication and Interpersonal skills

- Assess a learner’s ability to build rapport with patients.
- Assess a learner’s English proficiency.

Medical History

- Assess the way a learner gathers the history.

| SETTING: outpatient, in patient, ED, home, nursing home, rehab, group etc. | Outpatient/ Urgi Center |
| --- | --- |
| PATIENT PROFILE: Information about the “patient” that helps select an SP and helps the learner get an understanding of them as a person. SP will know more information about the patient than learner will ever ask but allows SP to portray a fully developed patient personality. If none of the items below are particulars for the case please write “all may be used.” | |
| Age range | 20-40 years old |
| Religious/spiritual background | All may be used |
| Sex (e.g., male, female, intersex, transwoman, transman) | All may be used |
| Sexual Orientation (e.g., heterosexual, lesbian, gay, bisexual, pansexual, queer, asexual) | All may be used |
| Gender expression (e.g., man, woman, gender queer) | All may be used |
| Race/ethnicity: | All may be used |
| Physical description (e.g., BMI, height range) | All may be used |
| Physical limitations | None |
| Patient appearance (e.g., disheveled, hospital gown, business casual, casual) | Hospital gown |
| Moulage + location (e.g., none, bruises, scars, body piercing, tattoos) | Legs |
| Affect (e.g., pleasant, cooperative) | Pleasant and cooperative |
| Family group (e.g., who is family, who they live with) | Not applicable |
| Education | College level |
| Level of health literacy | All may be used |
| Employment, if any - present and past, noting any current stresses | Full time Kindergarten teacher |
| Home/homeless - type of dwelling, number of stories, owned or rented | Home-owner |
| Financial situation- any current stresses | No stress- employed |
| Insurance Status (e.g., un/under/insured, public/private, HMO/PPO) | Insured through job |
| Habits (i.e., diet, exercise, caffeine, smoking, alcohol, drugs) | Smoking: you never smoked  Coffee/Caffeine: you drink a coffee in the morning – and 1-2 teas in the afternoon. In the summer you drink ice tea. You occasionally have a coke – but not often  Alcohol/Drugs: You have a glass of wine only on the weekends if you go out to dinner. No drugs.  Nutrition: You try to eat a well-balanced and healthy diet.  Exercise: no formal exercise program. Walks the dog ½ mile a day every morning and evening.  Sleep: you get a well-rested full night’s sleep normally. |
| Activities (i.e., hobbies, sports, clubs, friends) | Not applicable |
| Typical day - what is the usual daily routine | Not applicable |
|  |  |
|  |  |

| CASE INFORMATION | |
| --- | --- |
| Chief Concern: What the patient will say when greeted by the student. The patient’s primary reason for seeking medical care often stated in his/own words. | I am here to get antibiotics for my sinus infection |
| Additional Concerns: Other, if any, concerns the patient has today (i.e., symptoms, requests, expectations, etc.) that will become part of set agenda. | none |
|  | |
| THE PATIENT STORY: The SP will be asked to tell their symptom story and the personal and emotion impact for each of their concerns. You will want to write this is the patient voice. The symptom story should be able to answer this question: “Tell me more about [chief concern/additional concern], starting at the beginning and bringing me up to now.”  The personal context should be able to answer questions concerning the broader personal/psychosocial context of symptoms, especially the patient beliefs/attributions.  The emotional context should be able to ask how are you doing with this, how does this make you feel, how has this affected you emotionally? IMPACT: How has this affected your life? How has this been for your family? | Patient Story: Tell me more response  “I started not to feel right three days ago. At first just a bit under-the weather – tired, a bit achy and a slight headache. I took some vitamin C and went to bed early and tried to sleep it off. However, I wasn’t any better in the morning. My head hurt even more, and I felt a lot of pressure in my facial sinuses and in my ears, especially on the right side. It almost feels like I like I am underwater. My right ear hurts inside – like a pressure. I took some decongestant but yesterday my temperature popped up to 102 and when I was blowing my nose all this thick yellow to greenish stuff was coming out. Just feels like a typical sinus infection – I don’t want it to go into my lungs and now I am not short of breath at all and I don’t feel it in my lungs, so I want to get some antibiotics before it spreads there!”  Patient Story: Back story  You went to a Northwell GoHealth Urgent Care center for what seems like a fairly typical sinus infection. You get sinus and ear infections 2-3 times a year. Sometimes it even goes into pneumonia. You have had pneumonia three times in the last 10 years – and that is with the pneumonia vaccine.  Other than this infection you consider yourself healthy. The history of bronchiectasis is relatively recent (last bout of pneumonia was last year, at which time your primary doctor said you also had this). You cough up every day with thick and light-yellow phlegm but occurs usually once in the morning when you first wake up. You have a bout of coughing, bring up this phlegm and then go about your day. You have only noticed shortness of breath when you exert yourself, like running up the stairs.  Personal context: explanatory model  “I am constantly exposed to sniffly, sick kids at work”  Emotional context: impact on life  “if I get my antibiotics I do fine and there is no impact. If I don’t get my antibiotics, then I get sick really fast.”  Emotional context: impact on family  Any may be used  Emotional context: concern  “I am concerned that if I don’t get antibiotics quickly, I will get really sick” |
| HISTORY OF PRESENT ILLNESS: Although some of the HPI will be given in the patient’s symptom story, the learners will expand the story during the direct question section. Below describe the detailed history, usually about the chief concern, which the student must develop in order to make a useful assessment of the problem: | |
|  | |
| Onset (when; gradual or sudden) | Gradual |
| Setting (what was going on or where was patient when symptoms first noticed?) | All may be used |
| Duration (how long) | Three days |
| Time relationships (frequency, constant or intermittent) | Constant |
| Location | Facial sinuses, headache, right ear |
| Radiation | None |
| Quality | Achy |
| Amount | Moderate amount of thick sputum |
| Aggravated by what | Nothing |
| Relieved by what | Nothing |
| Associated with what | Fever of 102 |
| Attitude (what does the patient think is the problem, and how does he/she feel about it) | Early sinus infection  Very typical |
| Overall course | Not improving |
| REVIEW OF SYSTEMS: Significant positives and negatives | |
| Fever | 102 F |
| Mucous | Thick yellow to green |
| Cough/Sputum | Not more than my chronic cough and sputum production (which is a daily, thick yellow sputum, usually upon awakening in the morning |
| Shortness of breath | No |
| Chest pain | No |
| Blood in the sputum or bloody nasal discharge | No |
| Weight loss | No |
| Kidney problems or changes in urine | No |
| Rashes | Yes (moulage). Lately I have noticed little red dots on my legs and arms, particularly when holding bags of groceries. They don’t itch, they don’t hurt, and they don’t seem to be getting worse. |
| Diarrhea | Yes, watery stools daily. No blood. |
| Lymph node enlargement | No |
|  |  |
|  | |
| Past medical history | Bronchiectasis (early)  Bronchitis at least once a year  Pneumonia, 3x in the lst 10 years |
| Medication allergies (Name and reaction) | None |
| Environmental allergies (Name and reaction) | None |
| Illnesses | Frequent sinus, bronchitis and pneumonia since age of 17 |
| Vaccinations | Flu shot yearly in November  Received all childhood vaccines  Pneumonia vaccine (several years ago – doesn’t remember when) |
| Surgeries | None |
| Accidents/ injuries/ trauma | None |
| Hospitalization | None |
|  | |
| Inclusive sexual and reproductive history | |
| Sexual practices  Sexual partners  Protection: Use of safer sex practices  Use of birth control if appropriate  Risk of intimate partner violence | Not applicable |
| Ob/GYN HISTORY | Age of onset of menses- All may be used  Age of menopause- All may be used  Number of pregnancies- All may be used  Number of live births- All may be used  Number of miscarriages- All may be used  Number of abortions- All may be used |
| Medications | Prescription/dose/reason- no current medications  Over the counter/dose/reason- no current OTC medications  Herbs/supplements/dose/reason- no current supplements  Other: NOTE - All may be used |
| Immunizations | 🗹 Tetanus  🗹 Flu  🗹 Hepatitis  🗹 Pneumovax   - HPV - Other |
| Tobacco products:   - Cigarettes - Cigar - Pipe - Chew - E-cigarettes | 🗹 Never   - Past- year started/year quit - Current   - Quantity   - # of years |
| Alcohol   - Beer - Wine - Liquor - Other | - Never - Past- year started/year quit   🗹 Current   - - Quantity – wine on weekends occasionally   - # of years – all may be used |
| Drugs   - Weed - Cocaine - Heroin - Meth - Other - IV - Inhalants - Other | 🗹 Never   - Past- year started/year quit - Current   - Quantity - # of years |
| Diet (describe) | Not applicable |
| Exercise (describe) | Not applicable |
| List any other important social history or information important to this case | Not applicable |
| Family history |  |
| Mother, Father, Siblings, Grandparents, and other significant findings. | No history of infections |
|  |  |
| Physical Exam- List exam maneuvers expected for this case and any abnormal findings that SP will simulate. (tenderness, hyper-hypo reflex, rebound, weakness etc. )  Observation: You can perform all required physical exam maneuvers without difficulty. You breathe comfortably throughout the exam without exertional dyspnea.  Vital Signs: nothing to simulate  HEENT: If student performs otoscopic exam, hand them the ear PE finding card.  Sinuses: some tenderness over the eyes and forehead with pressure of sinuses  Skin: Moulage will be applied to your lower legs. It will be a diffuse small scattered erythematous macule. As the student examines your rash, say “isn’t that weird, it’s so flat that you can’t even feel it!”  Neck: nothing to simulate  Lungs You will have SimScope patches placed on your front and back lung fields  Heart You will have SimScope patches placed over your heart  Abdomen: You will have a SimScope patch on your belly  Extremities: If student examines the nailbeds, hand them the finger PE finding card | |
| PHYSICAL EXAM FINDINGS |  |
| 1. Written in layman’s terms |  |
| 1. General appearance- affect, appearance, position of patient at opening (i.e. sitting, laying down, holding abdomen etc.) | Well groomed, sitting comfortably, able to comply with physical exam without exertional dyspnea |
| 1. Vital signs | Nothing to simulate |
| 1. Specific findings and affect | Some tenderness over sinuses |
| 1. Response to certain physical movements | If student examines the skin, say “isn’t that weird, it’s so flat that you can’t even feel it” |
|  |  |
| DIAGNOSIS AND DIFFERENTIAL |  |
| Diagnosis with support from positive and negative history and PE findings | Sinusitis due to underlying primary immunodeficiency (common variable immunodeficiency)   - 1. historical support:      1. recurrent sinopulmonary infections that began after childhood      2. poor response to immunizations      3. bronchiectasis      4. diarrhea      5. increased bruising (for associated autoimmune ITP)   2. pe support:      1. otitis media (in adult),      2. clubbing (in support of bronchiectasis)      3. purpura (possibly due to ITP as it blanches without palpable component making vasculitis much less likely). |
| Differential with support from positive and negative history and PE findings | Vasculitis such as Wegener’s granulomatosis:   - 1. historical support:      1. systemic disease, affecting multiple organs      2. characteristic pattern of involvement upper respiratory (currently sinus) and lower pulmonary disease      3. history of rash   2. pe support:      1. Otitis media (in adult),      2. petechial/purpuric rash   Recurrent sinopulmonary infections from disease that result in impaired airway clearance (cystic fibrosis or ciliary dyskinesia)   - 1. historical support:      1. systemic disease, affecting multiple organs      2. purulent sputum from bronchiectesis   2. pe support:      1. Otitis media (in adult), |
|  |  |
| MANAGEMENT OR DIAGNOSTIC PLAN | The student is not asked to create a management plan for this patient, however, the plan would be:   1. sinusitis: treat with antibiotics 2. underlying diagnostic workup: assess patient for immunodeficiency with immunoglobulins, T-cell and B-cells, and functional response to vaccinations (titers). Assess the patient for vasculitis with serologic testing including ANCA and ESR. |
|  |  |
| PROFESSIONALISM ISSUES OR CHALLENGES: | The challenge in this case is to recognize the pattern of repeated sinus and pulmonary infections to identify an underlying etiology – immunodeficiency. |
