# Supplementary material for: Common Variable Immunodeficiency: A Standardized Patient Case for Second-Year Medical Students
Source: MedEdPORTAL. 2019 Oct 18;15:10837. doi: 10.15766/mep_2374-8265.10837 (PMC6974347; doi:10.15766/mep_2374-8265.10837)
Supplement: Supplementary file 1 — A. SP Case.docx B. SP Training Notes.docx C. PE Cards.docx D. Moulage.docx E. Door Chart and Instructions.docx F. Postencounter and Rubric.docx G. SP Checklist.docx [file mep-15-10837-s001.zip › B. SP Training Notes.docx]

Appendix B:  *SP Training Notes*

**Patient Name:** Robin Samuels

Setting: Outpatient/ Ambulatory Urgi-Center

Chief Complaint: “I’m here to get antibiotics for my sinus infection”

**Patient Profile**

SP recruitment profile: 20-40 years old, adaptable to any sex, race and age

Appearance: well-groomed, hospital gown

Moulage: petechiae, lower extremities (appendix INSERT)

Affect: Pleasant and cooperative, normally a high energy person

Employment: Full time kindergarten teacher in Elmhurst, NY. You love your job and cannot think of any other job that you would want to have.

Education: You graduated high school and college – you were working on your masters but couldn’t keep up with it between your children, work and getting sick so often…. You decided to hold off on it for a few years until your children are a bit older.

Lifestyle: You lead a simple yet busy life, particularly as you balance working full time at a school and taking care of your three young children. They are involved in many after school activities and you love taking them to all of them. Your husband works in his own business (restaurant) and so is not around that much – weekends and evenings are his busy times and so you must do most of the child care.

Relationships: You have been married to your high school sweetheart for 15 years. You have a strong relationship. You have never been unfaithful to him, nor do you believe he has to you.

Habits: Smoking: you never smoked

Coffee/Caffeine: you drink a coffee in the morning – and 1-2 teas in the afternoon. In the summer you drink ice tea. You occasionally have a coke – but not often

Alcohol/Drugs: You have a glass of wine only on the weekends if you go out to dinner. No drugs.

Nutrition: You try to eat a well-balanced and healthy diet.

Exercise: no formal exercise program. Walks the dog ½ mile a day every morning and evening.

Sleep: you get a well-rested full night’s sleep normally.

Religion: You are protestant and attend service regularly

**Case information**:

Chief complaint: “I’m here to get antibiotics for my sinus infection”

Response to “tell me more”: “I started not to feel right three days ago. At first just a bit under-the weather – tired, a bit achy and a slight headache. I took some vitamin C and went to bed early and tried to sleep it off. However, I wasn’t any better in the morning. My head hurt even more, and I felt a lot of pressure in my facial sinuses and in my ears, especially on the right side. It almost feels like I like I am underwater. My right ear hurts inside – like a pressure. I took some decongestant but yesterday my temperature popped up to 102 and when I was blowing my nose all this thick yellow to greenish stuff was coming out. Just feels like a typical sinus infection – I don’t want it to go into my lungs and now I am not short of breath at all and I don’t feel it in my lungs, so I want to get some antibiotics before it spreads there!

Additional concerns/agenda: none

History of Present Illness: You went to a Northwell GoHealth Urgent Care center for what seems like a fairly typical sinus infection. You get sinus and ear infections 2-3 times a year. Sometimes it even goes into pneumonia. You have had pneumonia three times in the last 10 years – and that is with the pneumonia vaccine.

Other than this infection you consider yourself healthy. The history of bronchiectasis is relatively recent (last bout of pneumonia was last year, at which time your primary doctor said you also had this). You cough up every day with thick and light-yellow phlegm but occurs usually once in the morning when you first wake up. You have a bout of coughing, bring up this phlegm and then go about your day. You have only noticed shortness of breath when you exert yourself, like running up the stairs.

ONLY IF ASKED: You have had many sinus and ear infections since you were about 18 years old. You have even had pneumonia so many times that you have some permanent lung damage from it [bronchiectasis]. You attribute this to your job as a kindergarten teacher because as a kid, you were always very healthy, rarely got colds and didn’t even have tubes in your ears.

ONLY IF ASKED - Are you short of breath? Not more than I usually am. My bronchiectasis is mild and generally only get short of breath if I am exerting myself, likely walking up a couple of flights of stairs.

ONLY IF ASKED- Are you coughing more? My cough is generally mild but daily. It is not worse than it has been.

ONLY IF ASKED- Do you have chest pain? No

ONLY IF ASKED- Do you have cough up blood? No

ONLY IF ASKED- Is your nasal discharge bloody? No

ONLY IF ASKED- Have you had weight loss? No

ONLY IF ASKED- Are you bringing up more phlegm than usual? no

ONLY IF ASKED- Do you have any kidney problems? No

ONLY IF ASKED- Do you have any rashes? Lately you notice these little red dots on your legs and arms where you were holding some groceries. They don’t itch, they don’t hurt, they don’t seem to be getting worse and they fade. You weren’t that concerned about them. during the PE if the student is examining it – say “isn’t that weird – it is so flat you can’t feel it.

Impact on Life: “if I get my antibiotics I do fine and there is no impact. If I don’t get my antibiotics, then I get sick really fast.”

Concern: “you are concerned that if you don’t get antibiotics quickly you will get really sick”

Explanatory model: “You are constantly exposed to sniffly, sick kids at work.”

Review of systems: Do not volunteer these symptoms – only disclose them if asked

Pertinent Positives:

- With the bronchiectasis you have a chronic cough with mucous production – there has been no change in this with this infection.
- You have chronic diarrhea – with watery stools daily – no hx of gi infections like salmonella, campylobacter or giardia. You attribute this to irritable bowel syndrome (IBS).
- Lately you seem to be bruising a bit more easily- you seem to get them out of nowhere – they are mostly on your legs, but you also noticed them on your arm where you were holding a bag of milk.

Pertinent Negatives:

- No weight loss
- No history of cancers or lymphomas
- No history of lymph nodes being enlarged

Past medical history: Early bronchiectasis.

You did NOT have a lot of infections during your childhood. You began having recurrent sinusitis that started in your late teems – around 17 or 18. It often goes into your chest and you develop bronchitis – about once a year. You have even had pneumonia 3 x in the last 10 years – last year it was “pretty bad”. You recently have been worked up and told you have bronchiectasis.

Medications: no prescription, over the counter or herbal medications.

Allergies : denies medication, environmental or food allergies

Vaccinations: Flu shot yearly in November

Received all childhood vaccines

Pneumonia vaccine several years ago at PMD – “was supposed to help with my sinus issues and prevent me from having pneumonia again, but apparently it didn’t work out too well”

Sexual hx: Your husband is your one and only lifetime partner.

Family hx: Mother. Alive-age 76. High blood pressure, high cholesterol

Father. Alive-age 77. Gout

Physical Exam

General: You are well groomed, in a gown on the exam table. You can perform all required physical exam maneuvers without difficulty. You breathe comfortably throughout the exam without exertional dyspnea.

Vital Signs:

HEENT: If student performs otoscopic exam, hand them the ear PE finding card

Skin: Moulage will be applied to your lower legs. It will be a diffuse small scattered erythematous macule. As the student examines your rash, say “isn’t that weird, it’s so flat that you can’t even feel it!”

Lungs You will have SimScope patches placed on your front and back lung fields (rhonchi in lower fields)

Heart You will have SimScope patches placed over your heart (normal)

Abdomen: You will have a SimScope patch on your belly (normal)

Extremities: If student examines the nailbeds, hand them the finger PE finding card
