# Supplementary material for: Common Variable Immunodeficiency: A Standardized Patient Case for Second-Year Medical Students
Source: MedEdPORTAL. 2019 Oct 18;15:10837. doi: 10.15766/mep_2374-8265.10837 (PMC6974347; doi:10.15766/mep_2374-8265.10837)
Supplement: Supplementary file 1 — A. SP Case.docx B. SP Training Notes.docx C. PE Cards.docx D. Moulage.docx E. Door Chart and Instructions.docx F. Postencounter and Rubric.docx G. SP Checklist.docx [file mep-15-10837-s001.zip › C. PE Cards.docx]

Appendix C:  *PE Cards*

If otoscopic exam is performed:

3x 5 index card with image of otitis media

If hands and nails are examined:


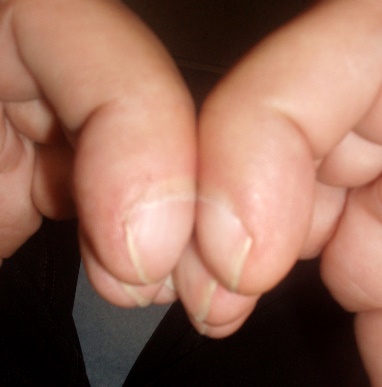
3x5 index card with image of clubbing*

*Image retrieved from: [https://meded.ucsd.edu/clinicalimg/upper_clubbing.htm on 1.5.19](https://meded.ucsd.edu/clinicalimg/upper_clubbing.htm%20on%201.5.19)

Permission received from Charles Goldberg, MD.
