# Supplementary material for: Common Variable Immunodeficiency: A Standardized Patient Case for Second-Year Medical Students
Source: MedEdPORTAL. 2019 Oct 18;15:10837. doi: 10.15766/mep_2374-8265.10837 (PMC6974347; doi:10.15766/mep_2374-8265.10837)
Supplement: Supplementary file 1 — A. SP Case.docx B. SP Training Notes.docx C. PE Cards.docx D. Moulage.docx E. Door Chart and Instructions.docx F. Postencounter and Rubric.docx G. SP Checklist.docx [file mep-15-10837-s001.zip › D. Moulage.docx]

Appendix D:  *Moulage*

Time:

3-5 minutes

Supplies:

1. Light skin tones: Moulage concepts Inc, Paradise, CA: Trauma Spectrum Color Stix Makeup Stix
   1. CL-15 (maroon)
   2. CL-18 (purple)
2. Medium skin tones: Ben Nye Crème Color
   1. CL-15 (maroon)
   2. FX-5 (dark burgundy)
3. Dark skin tones: Ben Nye Crème color
   1. CL-15 (maroon)
   2. CL-17 (Misty Violet)
4. Medium to dark skin tones: Ben Nye Final Seal
5. Porous sea sponge
6. Q-tips

Technique:

Apply moulage to sea sponge, using a spritz of water to activate the color. Dot the sponge lightly over the area of skin to create a stipulated effect. Using the wooden end of a Q-tip, apply dots of darker color throughout the sponged-on makeup. Finally, the moulage on darker skin tones was sprayed with sealant. The Makeup Stix product used on fairer skin tones seals on its own after drying and therefore there was no need to apply an extra sealant.

Color Palette:

Color palette and hue were adjusted for skin tone.

Examples:

Figure 1. light skin tone

Figure 2. Medium skin tone

Figure 3. Dark skin tone

*All figures are author owned
