# Supplementary material for: Common Variable Immunodeficiency: A Standardized Patient Case for Second-Year Medical Students
Source: MedEdPORTAL. 2019 Oct 18;15:10837. doi: 10.15766/mep_2374-8265.10837 (PMC6974347; doi:10.15766/mep_2374-8265.10837)
Supplement: Supplementary file 1 — A. SP Case.docx B. SP Training Notes.docx C. PE Cards.docx D. Moulage.docx E. Door Chart and Instructions.docx F. Postencounter and Rubric.docx G. SP Checklist.docx [file mep-15-10837-s001.zip › E. Door Chart and Instructions.docx]

Appendix E:  *Door Chart and Instructions*

PATIENT NAME: Robin Samuels

DOB: October 4^th^, 1988

Robin Samuels is here at the INSERT INSTITUTIONAL NAME Urgent Care center for an urgent visit.

Vital Signs:

HR: 94

RR: 18

BP: 108/74

Temp 102 F

O2 on Room Air: 96%

You are to:

1. Elicit a chief complaint
2. Gather the pertinent history
3. Perform a hypothesis driven physical exam

You do NOT need to repeat vital signs

Encounter time: 15 minutes

Postencounter time: 10 minutes
