# Supplementary material for: Common Variable Immunodeficiency: A Standardized Patient Case for Second-Year Medical Students
Source: MedEdPORTAL. 2019 Oct 18;15:10837. doi: 10.15766/mep_2374-8265.10837 (PMC6974347; doi:10.15766/mep_2374-8265.10837)
Supplement: Supplementary file 1 — A. SP Case.docx B. SP Training Notes.docx C. PE Cards.docx D. Moulage.docx E. Door Chart and Instructions.docx F. Postencounter and Rubric.docx G. SP Checklist.docx [file mep-15-10837-s001.zip › F. Postencounter and Rubric.docx]

Appendix F:  *Postencounter and Rubric*

Question:

What two conditions are on your differential in order of most to least likely to explain Ms. Samuels condition?

(BE AS SPECIFIC AS POSSIBLE!). Defend EACH diagnosis with at least one historical and physical evidence.

Ideal Answer:

1. Sinusitis due to underlying primary immunodeficiency (common variable immunodeficiency)
   1. historical support:
      1. recurrent sinopulmonary infections that began after childhood
      2. poor response to immunizations
      3. bronchiectasis
      4. diarrhea
      5. increased bruising (for associated autoimmune ITP)
   2. pe support:
      1. otitis media (in adult),
      2. clubbing (in support of bronchiectasis)
      3. purpura (possibly due to ITP as it blanches without palpable component making vasculitis much less likely).
2. Sinus and pulmonary disease from vasculitis (Granulomatosis with polyangiitis)
   1. historical support:
      1. systemic disease, affecting multiple organs
      2. characteristic pattern of involvement upper respiratory and lower pulmonary disease
      3. history of rash
   2. pe support:
      1. Otitis media (in adult),
      2. petechial/purpuric rash

Rubric:

Total points 11

1. Identification of specific (CVID or IgA deficiency) primary immunodeficiency as a cause of recurrent sinopulmonary infections (2 points) – only 1 point for PIDD without specific type or incorrect type
   1. Historical support (1 point)
   2. PE support (1 point)
   3. Depth of explanation (1 point)
   4. Prioritization (1 point)
2. Granulomatosis with polyangiitis (2 points) – only 1 point for general vasculitis
   1. Historical support (1 point)
   2. PE support (1 point)
   3. Depth of explanation (1 point)

Partial credit given for reasonable alternative diagnosis – such as sinusitis (without recognizing underlying predisposing immunodeficiency), otitis or disease that causes impaired airway clearance (cystic fibrosis or ciliary dyskinesia).
