# Supplementary material for: Common Variable Immunodeficiency: A Standardized Patient Case for Second-Year Medical Students
Source: MedEdPORTAL. 2019 Oct 18;15:10837. doi: 10.15766/mep_2374-8265.10837 (PMC6974347; doi:10.15766/mep_2374-8265.10837)
Supplement: Supplementary file 1 — A. SP Case.docx B. SP Training Notes.docx C. PE Cards.docx D. Moulage.docx E. Door Chart and Instructions.docx F. Postencounter and Rubric.docx G. SP Checklist.docx [file mep-15-10837-s001.zip › G. SP Checklist.docx]

Appendix G:  *SP checklist*

Interpersonal and communication skills/Function 1 Building Rapport

1. Did the student refrain from exhibiting any distracting non-verbal behaviors? (i.e. flat affect, eye contact, posture, playing with hair, pen clicking, space building, swaying on stool, etc.)
   1. 0= No (please comment on specific behavior
   2. 2=Yes
2. Did the student use the skills of empathy in relation to an expressed or potential feeling/emotion (reflection, legitimation, exploration)
   1. 0=Did not se any of the skills of empathy
   2. 1=Used one skills of empathy
   3. 2=Used two or more skills of empathy
3. Did the student elicit the impact on your life, your concerns or your explanatory model? (“what do you think is going on?” or “Why do you think this happened?”)
   1. 0=Not at all
   2. 1=Partially (at least 1)
   3. 2=Completely (greater than 1)
4. Did the student update you as to what to expect during the physical exam?
   1. 0=No
   2. 1=Yes, but too much information was given
   3. 2=Yes

Interpersonal and communication skills/English proficiency

1. Is the student proficient in English? (syntax, word choice)
   1. 0=No (please comment)
   2. 1=Yes
2. Was the student’s articulation understandable? (fluency, clarity, pronunciation)
   1. 0=No (please comment)
   2. 1=Yes

Interpersonal and communication skills/Patient Care/Function 2 Gathering the history

1. Did the student ask our chief complaint? (“What brings you in ?”)
   1. 0=No
   2. 1=Yes
2. Did the student use medical jargon without explain it?
   1. 0=Frequently
   2. 1=Occasionally
   3. 2=Never
3. Did the student sanitize their hands with soap and water or Purell, before an physical contact? (also prior to PE if applicable)
   1. 0=No (please comment)
   2. 1=Incorrectly (please comment)
   3. 2=Yes, or the student did not make any physical contact
4. Did the student review and summarize the gathered information from the history of present illness (HPI), at any time during the encounter?
   1. 0=No
   2. 2=Yes

Physical examination/technique

1. Did the student attend to your comfort and modesty during the physical exam?
   1. 0=No (please comment)
   2. 1=Somewhat (please comment)
   3. 2=Yes
2. Was the progression of the physical exam fluid?
   1. 0=No
   2. 1=Somewhat
   3. 2=Yes
3. Did the student perform all physical exam procedures on skin?
   1. 0=No
   2. 1=Incorrectly (please comment)
   3. 2-Yes, correctly (please comment if wearing gloves)

Clinical Reasoning/Developing a differential diagnosis

1. Did the student elicit that you have a long history of infections?
   1. 0=No
   2. 1=Yes
2. Did the student ask about infections during your childhood?
   1. 0=No
   2. 1=Yes
3. Did the student ask if you are short of breath?
   1. 0=No
   2. 1=Yes

Clinical Reasoning/Hypothesis Driven physical exam

1. Did the student examine your skin?
   1. 0=No
   2. 1=Yes
2. Did the student examine your nose?
   1. 0=No
   2. 1=Yes
3. Did the student look in your ears?
   1. 0=No
   2. 1=Yes
4. Did the student look in your mouth?
   1. 0=No
   2. 1=Yes
5. Did the student examine your neck for lymph nodes?
   1. 0=No
   2. 1=Yes
6. Did the student examine your lungs?
   1. 0=No
   2. 1=Yes
7. Did the student examine your spleen (by palpation or percussion of left upper abdomen or lower ribcage)?
   1. 0=No
   2. 1=Yes
8. Did the student examine your nails?
   1. 0=No
   2. 1=Yes

Professionalism

1. Did the student leave the room when the “end of the encounter” prompt was made?
   1. 0=No
   2. 1=Yes
2. Did the student avoid verbal or nonverbal judgmental behaviors?
   1. 0=No (please describe behavior in comment box)
   2. 1=Yes
3. Did the student convey sincerity?
   1. 0=Did not come across as sincere (please describe behavior in comment box)
   2. 1=I found myself questioning his or her sincerity (please describe behavior in comment box)
   3. 2=Came across as sincere
